# Chaotic time series prediction for glucose dynamics in type 1 diabetes mellitus using regime-switching models

**DOI:** 10.1038/s41598-017-06478-4

**Published:** 2017-07-24

**Authors:** Mirela Frandes, Bogdan Timar, Romulus Timar, Diana Lungeanu

**Affiliations:** 1 Department of Functional Sciences, “Victor Babes” University of Medicine and Pharmacy, Timisoara, Romania; 2 Department of Internal Medicine, “Victor Babes” University of Medicine and Pharmacy, Timisoara, Romania; 3 “Pius Brinzeu” Emergency Hospital, Timisoara, Romania

## Abstract

In patients with type 1 diabetes mellitus (T1DM), glucose dynamics are influenced by insulin reactions, diet, lifestyle, etc., and characterized by instability and nonlinearity. With the objective of a dependable decision support system for T1DM self-management, we aim to model glucose dynamics using their nonlinear chaotic properties. A group of patients was monitored via continuous glucose monitoring (CGM) sensors for several days under free-living conditions. We assessed the glycemic variability (GV) and chaotic properties of each time series. Time series were subsequently transformed into the phase-space and individual autoregressive (AR) models were applied to predict glucose values over 30-minute and 60-minute prediction horizons (PH). The logistic smooth transition AR (LSTAR) model provided the best prediction accuracy for patients with high GV. For a PH of 30 minutes, the average values of root mean squared error (RMSE) and mean absolute error (MAE) for the LSTAR model in the case of patients in the hypoglycemia range were 5.83 ( ± 1.95) mg/dL and 5.18 ( ± 1.64) mg/dL, respectively. For a PH of 60 minutes, the average values of RMSE and MAE were 7.43 ( ± 1.87) mg/dL and 6.54 ( ± 1.6) mg/dL, respectively. Without the burden of measuring exogenous information, nonlinear regime-switching AR models provided fast and accurate results for glucose prediction.

## Introduction

Diabetes-related risk management includes prevention of both hypo- and hyper-glycemic episodes by maintaining safe blood glucose levels over time. In most cases, the management of type 1 diabetes mellitus (T1DM) is performed by the patient themselves. This is accomplished by using finger-stick blood glucose measurements, taken several times a day, with accompanying doses of rapid-acting and long-acting insulin based on the glycemic values, carbohydrate intake, physical activity, and several other physiological factors^1^. This can be very inconvenient when considering dynamic and complex conditions such as T1DM. Risk management may be compromised by lack of data and, in some cases, by the inability to adequately interpret data. Recent technologies, such as continuous glucose monitoring (CGM) sensors, measure glucose concentrations in subcutaneous (s.c.) interstitial space, providing valuable insights into glucose dynamics^2^. Coupled with rapid-acting insulin analogues delivered by insulin-pumps at a variable pace throughout the day, CGM sensors enable on-line decision support, e.g. closed-loop control systems or an “artificial pancreas”^3^. One critical part of the system is the algorithm for calculating the insulin delivery rate based on glucose measurements.

Glycemic variability (GV) is the degree to which a patient’s blood glucose fluctuates between high and low levels. This measure provides a comprehensive view of postprandial glycemic events and episodes of hypo- and hyper-glycemia^4^. Assessment of GV aids in determining how daily actions impact hypo- and hyper-glycemic events by associating out-of-target glucose levels with patient-specific factors, such as activity, food, stress, illness, and medication^5, 6^.

Chaotic time series prediction has been widely applied to various domains, such as signal processing, economics, and medicine^7^. The dynamic behaviour of a chaotic deterministic system is investigated by using univariate time series, which enable prediction of a chaotic sequence over short time-intervals. Kroll^8^ observed that biological variations in glucose and insulin levels include a deterministic chaotic component. Furthermore, identifying chaotic behaviour in glucose profiles may lead to an improved understanding of glucose dynamics in T1DM and facilitate alternative strategies for automatic control^9^. Demonstrable chaotic features include sensitivity to initial conditions, i.e. the system’s trajectory may diverge based on different configurations of similar starting conditions, and dependence on close monitoring^10^. Chaotic systems have a strange attractor that generates directional flows and circular movements, i.e. trajectories often diverge, but return to the same area after enough time has passed^8^. Directional movements imply deterministic behaviour, which is important because this ensures the power of prediction.

Prediction of glucose values based on CGM measurements allows the patient to make therapeutic decisions based on expected future glucose levels, rather than the current levels, thus decreasing the risk of hypo- and hyper-glycemic events. CGM data was firstly analysed as a time-series formulation employing 10-minute sampled data intervals and autoregressive (AR) models^11^. The predictive horizons were 10, 20, and 30 minutes, with the 10-minute predictions being the most accurate. Other proposed methods applying linear models, e.g. autoregressive (AR) and AR moving average (ARMA) models, demonstrated short-term prediction capabilities^12^. Nonlinear approaches ranged from data-driven models^13^ and artificial neural network (NN) models^14, 15^ to support vector regressions^16^ and generalized AR conditional heteroscedasticity approaches^17^. However, the problem of glucose prediction has remained challenging due to a high degree of inter- and intra-patient biological variation, and high glucose variability.

When modelling glucose dynamics, we exploit the fact that glucose time series exhibit deterministic chaotic behaviour, and that the physiological interactions that occur are multidimensional, nonlinear, and specific to each patient. Additionally, we consider that the CGM signal itself contains structural information about the time-changes in glucose concentrations. Therefore, no additional levels of complexity are justified when a fast response is needed, as is the case in real-time decision support systems. Advantages include no additional effort or discomfort for the patient caused by wearing additional sensors and tracking events. To the best of our knowledge, this is the first work focusing on the predictive modelling of glucose dynamics based on the chaotic properties of glucose time series in conjunction with regime-switching predictive models. Regime-switching models are convenient for predicting time series with dramatic changes in their behaviour. Abrupt changes in relatively short time intervals are a common feature in glucose time series, especially for periods of high GV and high risk of hypo- or hyper-glycemia.

## Results

### Glucose data analysis

#### Risk assessment

Risk levels were computed via the *low glucose index* (LGI) and *high glucose index* (HGI), which are linked to the frequency and severity of hypo- and hyper-glycemic episodes, respectively^18–20^. The higher the LGI and HGI values, the more frequent or extreme the hypo- and hyper-glycemia episodes. For computing the indices, a nonlinear transformation of CGM data was applied first, and then the risks associated with a hypo- or hyper-glycemic event were derived. The risk corresponding to each CGM reading was summed up in the respective LGI and HGI indices. Table 1 presents the percentages of landmark time intervals at different risk levels of hypo- and hyper-glycemia.

**Table 1:** Percentages of patients at different risk levels of hypo- and hyper-glycemia when considering the entire monitoring period (n = 17).

Risk [%]	Landmark time intervals						
	M	LM	EN	AN	EE	E	N
*Hyperglycemia* ^(1)^							
Minimal	47.06	41.18	29.41	41.18	35.29	35.29	41.18
Low	11.76	23.53	29.41	5.88	23.53	23.53	23.53
Moderate	17.65	11.76	23.53	35.29	5.88	17.65	5.88
High	23.53	23.53	17.65	17.65	35.29	23.53	29.41
*Hypoglycemia* ^(2)^							
Low	47.06	41.18	41.18	35.29	52.94	41.18	52.94
Moderate	17.65	35.29	23.53	29.41	11.76	29.41	11.76
High	35.29	23.53	35.29	35.29	35.29	29.41	35.29

Abbreviations: M, Morning; LM, Late-morning; EN, Early-noon; AN, Afternoon; EE, Early-evening; E, Evening; N, Night. ^(1)^Risk levels are defined by the Low Glucose Index (LGI): minimal risk for hypoglycemia (LGI less than 1.1); low-risk (LGI between 1.1 and 2.5); moderate-risk (LGI between 2.5 and 5); high-risk (LGI greater than 5). ^(2)^Risk levels are defined by the High Glucose Index (HGI): low-risk (HGI between less than 4.5); moderate-risk (HGI between 4.5 and 9); high-risk (HGI greater than 9).

#### Variability

We analysed the glucose data by computing intra-day and inter-day GV along with the risk levels of hypo- and hyper-glycemia for each patient. GV was evaluated using several measures based on CGM readings: (a) intra-day indices, e.g. standard deviation (SD), coefficient of variation (CV), J-index, mean amplitude of glycemic excursions (MAGE), and continuous overall net glycemic action (CONGA); and (b) inter-day indices, e.g. mean absolute value of the differences between glucose values at the same time on two consecutive days (MODD)^21, 22^, glycemic variability index (GVI), and patient glycemic status (PGS)^6, 23^. Generally, SD should be no higher than mean/2 in T1DM^24^. The J-index interpretation is as follows: ideal control (10 to 20); good control (20 to 30); inadequate control (greater than 40). A higher CONGA value corresponds to greater glycemic variation. GVI values indicate: low variability, i.e. non-diabetic (1.0 to 1.2); modest variability (1.2 to 1.5); high variability (greater than 1.5). The median value of MAGE for patients with a high-risk of hyperglycemia ranged from 70 in the *afternoon* to 150 at *noon*. The MODD median value was approximately 140 during most of the landmark time intervals, except for the *evening*, when the maximum MODD value was nearly 350. The median J-index value reached 40, indicating inadequate glycemic control for patients at high-risk of hypoglycemia over all landmark time intervals. The GVI median value ranged from 2.2 for *night* to 4 for *noon*, corresponding to modest and high variability, respectively. The MAGE and GV measures for patients at high-risk of hypoglycemia were lower than those for patients at high-risk of hyperglycemia. We observed that the highest GV corresponds to patients at high-risk of hypo- and hyper-glycemia.

#### Chaotic properties

We investigated the glucose time series using nonlinear analytical methods, such as embedding space, correlation dimension, and Lyapunov exponents. The purpose of time delay embedding is to project the time series into a multidimensional phase-space that is representative of the original system^25^. Thus, we can investigate the dynamics of the original system by studying the dynamics of the system in the phase-space. The correlation dimension is a standard measure of the fractal dimensionality of an object embedded into a phase-space^26^. The correlation dimension of the glucose time series was 2.51 (±0.33) on average.

Each glucose time series yielded a positive Lyapunov exponent, although small in value (average of 0.21 ± 0.04). Thus, the glucose time series demonstrated deterministic chaotic behaviour, as shown by the existence of positive Lyapunov exponents.

**Figure 1:**
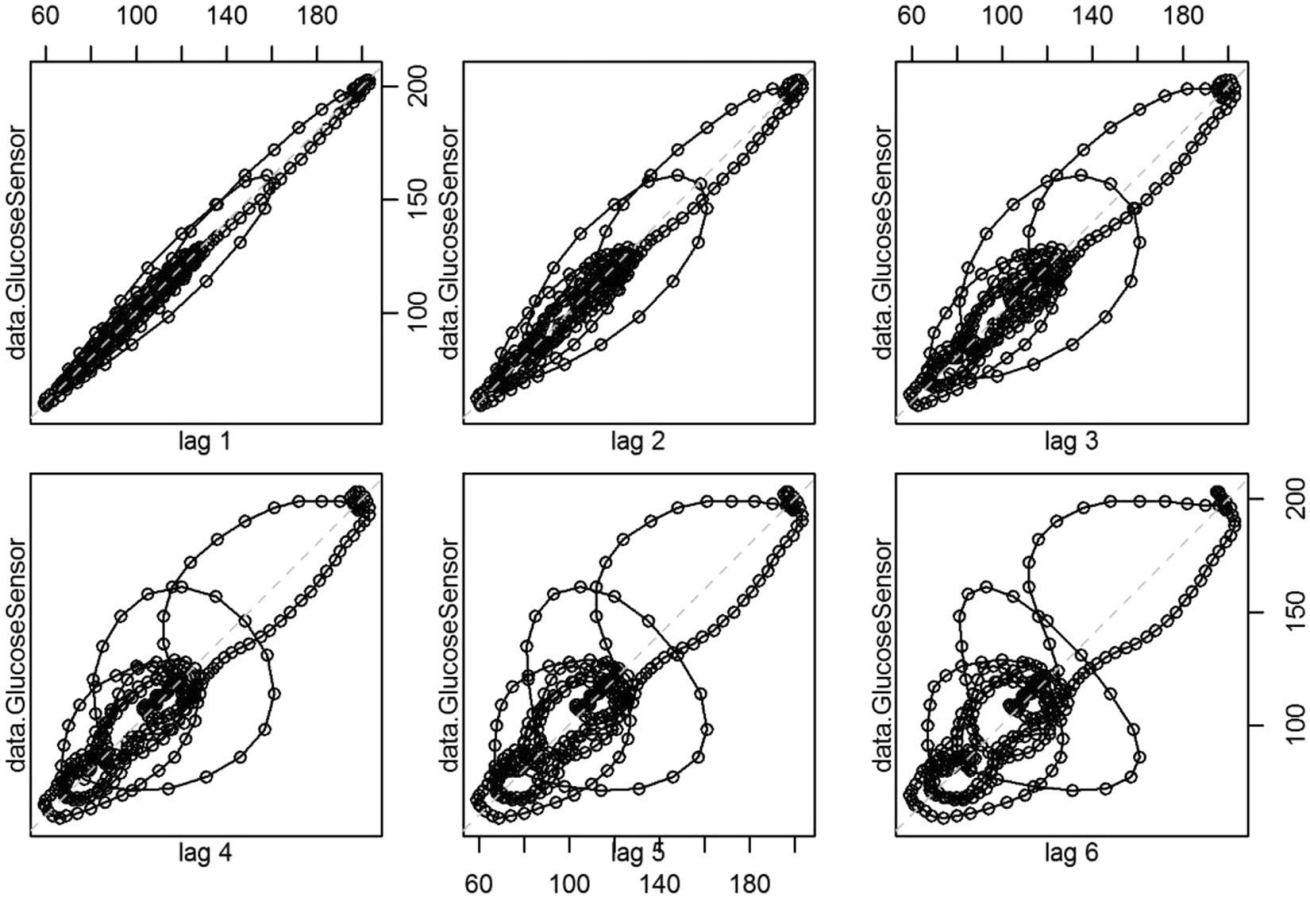
Projections of the phase portraits of glucose time series.

**Figure 2:**
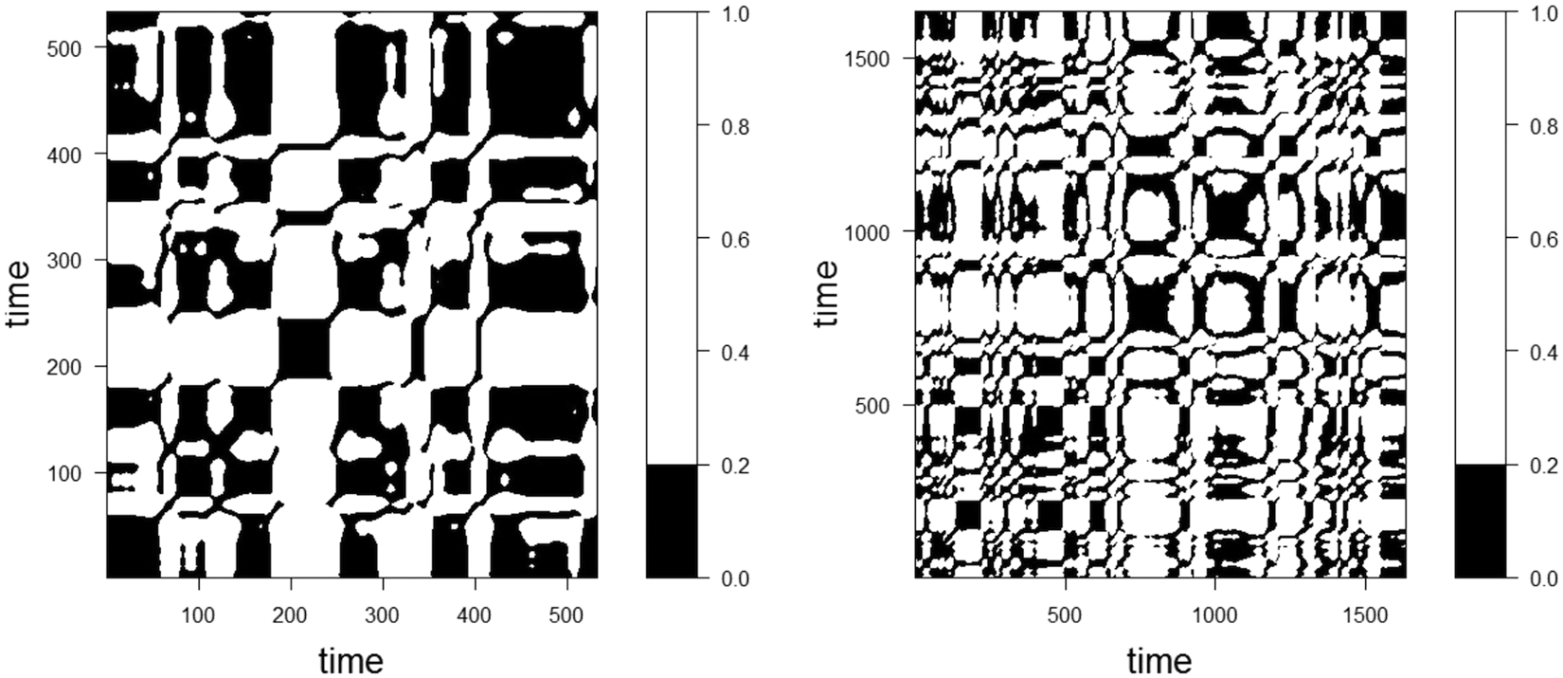
Recurrence plots of time series for landmark time intervals with a high risk of hypoglycemia (left) and a high risk of both hypo-and hyper-glycemia (right). Note that the plot is symmetric about the diagonal running from the lower left to the upper right.

Figure 1 presents projections of the phase portraits of glucose time series measured over 24 hours with different time lags. One can observe intense directional flows and circular movements, which implies the existence of a time-dependent behaviour exhibiting deterministic components. Figure 2 presents the recurrence plots for time series with a high risk of hypoglycemia and a high risk of both hypo-and hyper-glycemia, respectively. The recurrence plot is a 2-dimensional graph, with both axes corresponding to time. When the state space vectors corresponding to these time points are closer than a small cut-off distance, the point is coloured black in the graph; otherwise, no point is plotted. We observed deterministic cycles that originated from the embedding-reconstructed underlying dynamics.

Additionally, each time series was analysed using the Box-Jenkins methodology (Augmented Dickey-Fuller test of stationarity, model selection based on autocorrelation and partial autocorrelation functions, prediction diagnostics via Ljung-Box test) for linear models^26^. Data analysis was performed using the R project packages for statistical computing. A *p*-value of 0.05 was used as the statistical significance threshold.

### Time series prediction

#### Training and evaluation criteria

**Figure 3:**
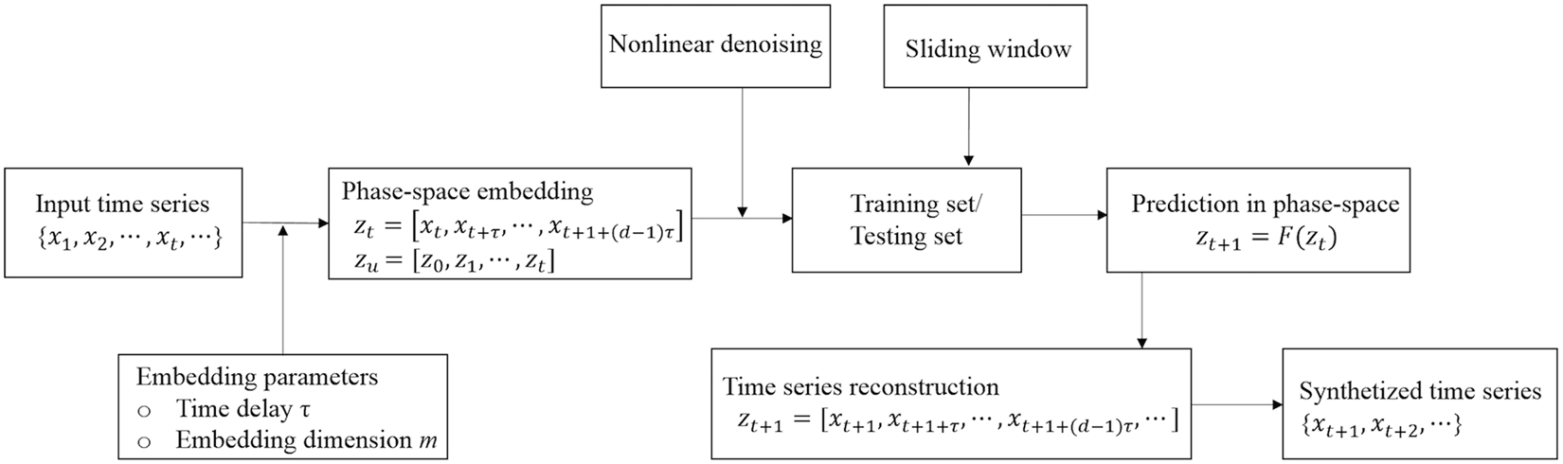
Main steps of time series prediction.

Patient-specific models were derived by capturing the chaotic properties of the corresponding glucose time series. For each PH, we used a training data set of eight monitoring hours, while the validation dataset was only used for performance evaluation. A sliding window approach was applied to continuously predict the glucose levels at preset PHs (30 minutes and 60 minutes ahead) using prior glucose data as the input for the AR models (Fig. 3).

Datasets underlying nonlinear dynamic behaviour typically contain noise, which is effectively random and displays no patterns in phase-space. Therefore, prior to entering data into the models, we performed a denoising step by averaging each Takens’ vector with its neighbours in an *m*-dimensional space when the time delay was 1. Each neighbourhood is specified within spheres of a given radius.

Time series reconstruction was performed by applying various regime-switching AR models, namely linear AR (LAR) models, additive AR (AAR) models, neural network based AR (NNAR) models, self-exiting threshold AR (SETAR) models, and logistic smooth transition AR (LSTAR) models. The least squares method was used to find the best fitting model by minimizing the sum of the squares of the residuals. The prediction performance of the linear and nonlinear AR models was evaluated by computing the root mean squared error (RMSE) and mean absolute error (MAE) of the out-of-sample predictions. Additionally, we performed continuous glucose-error grid analysis (CG-EGA)^27^ as a clinical evaluation criteria. The grid zones were defined as follows: zone A, no effect on clinical action; zone B, altered clinical action with little or no effect on clinical outcome; zone C, altered clinical action with possible effect on clinical outcome; and zone D, altered clinical action with possible significant medical risk.

#### Model parameters

**Figure 4:**
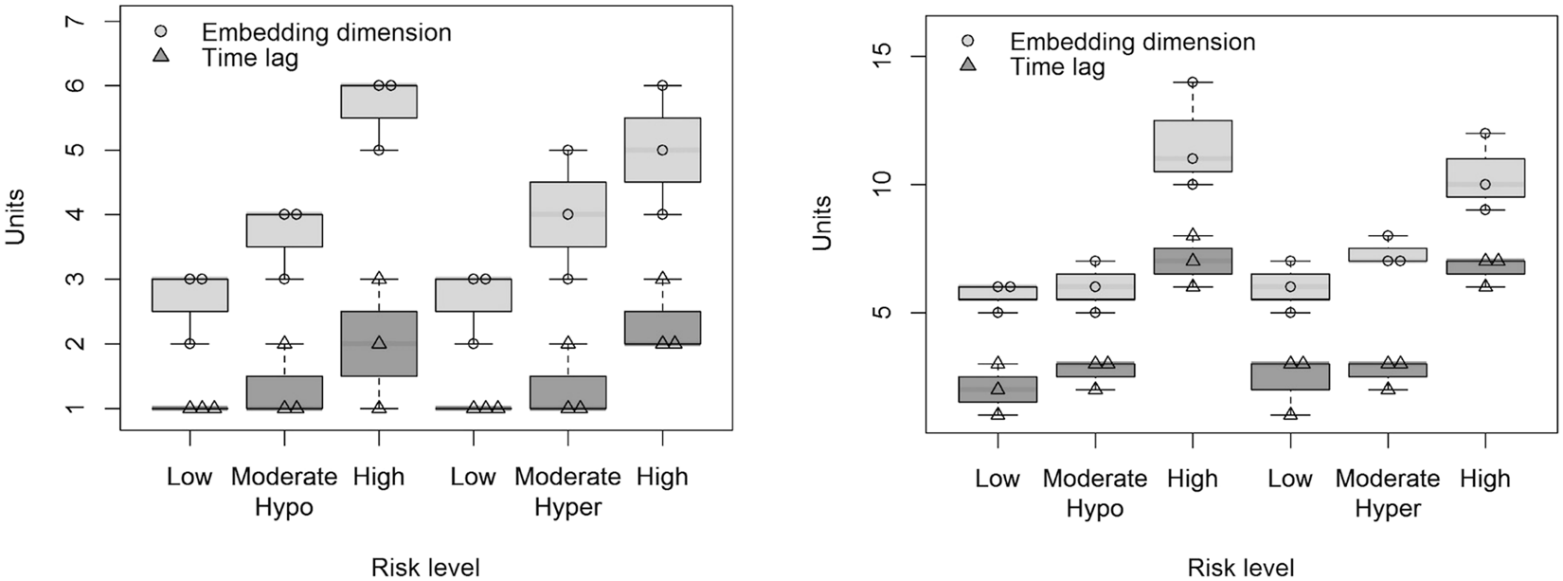
Embedding dimension *m* and time delay *τ* for time series of landmarks at different risk levels of hypo- and hyper-glycemia for a PH of 30 minutes (left) and 60 minutes (right).

The optimal embedding dimension *m* was chosen by using the false neighbours’ statistic and applying Cao’s algorithm^28^. Time delay *τ* was computed based on the autocorrelation function and the average mutual information of the corresponding landmark time intervals from the training dataset for linear and nonlinear models, respectively. We observed that both the embedding dimension and time delay varied based on the risk level of the corresponding landmark time intervals from the training dataset, presenting small values for low and moderate levels, and increased values for higher risk levels (Fig. 4).

The NN we used followed the structure of time-lagged feed-forward neural networks, including hyperbolic tangent functions in the hidden layers, as well as a linear function for the output layer. The NN was trained using the backpropagation algorithm. The number of hidden units *q* was selected automatically based on the embedding parameters (embedding dimension and time delay). The resulting hidden layer’s dimension was higher for patients with a high risk of hypo-and hyper-glycemia. The selection criteria for the optimal number of hidden units were the Akaike Information Criterion (AIC) and the Mean Absolute Percentage Error (MAPE).

The hyper-parameters for the SETAR models, namely AR order of the ‘low’ *L* and ‘high’ *H* regimes, and the threshold delay *δ*, were selected automatically based on the embedding dimension and time delay. The optimization criterion was the pooled-AIC. Here, both *L* and *H* were equal to *m* for all input time series. We chose the starting parameters for the LSTAR models by performing a two-dimensional grid-search over *c* and *γ*. The number of threshold values (*c*) in the grid was 200. The number of smoothing values (*γ*) in the grid was 40. The minimum percentage of observations in each regime was set to 10% (possible threshold values fell within the 0.1 and 0.9 quantiles). Estimation of the transition parameters was performed using the least squares method. The smoothing values of the grid were set to 1 and 40 for the lower and upper limit, respectively. The lower and upper threshold values of the grid fell between the 0.1 and 0.9 quantiles. The AR order for the ‘low’ regime *L* and the ‘high’ regime *H* should be less than or equal to the embedding dimension *m*.

#### Predictive modelling

The glucose time series were not stationary (Augmented Dickey-Fuller test, *p* < 0.05). Thus, the data was transformed in order to stabilize the variance and differentiated in order to obtain stationary series prior to predictive modelling. A denoising step was then applied, specifying the neighbourhood of Takens’ vectors within spheres of a given radius, ranging from 0.0001 for high embedding dimensions to 0.2 for low embedding dimensions. The applied predictive models were derived by selecting the best structure among all possible structures after the training process. Table 2 presents the average fitting quality of the five AR models for training on data sets of eight monitoring hours of a typical patient at high risk of both hyper- and hypo-glycemia, as well as other landmark time intervals for high and low-moderate risk levels.

**Table 2:** Average fitting quality of the five AR models (MAPE – *Mean Absolute Percentage Error*, AIC – *Akaike Information Criterion*).

Measure	Model				
	LAR	AAR	NNAR	SETAR	LSTAR
AIC	−1418.52 (230.11)	−1420.92 (211.54)	−1423.54 (462.62)	−1427.74 (233.81)	−1440.71 (224.65)
MAPE	0.0016 (0.00034)	0.0014 (0.00024)	0.0013 (0.00058)	0.0012 (0.00032)	0.0011 (0.00025)

Smaller values of AIC, BIC and MAPE correspond to higher quality models. Values are presented as mean (standard deviation).

**Figure 5:**
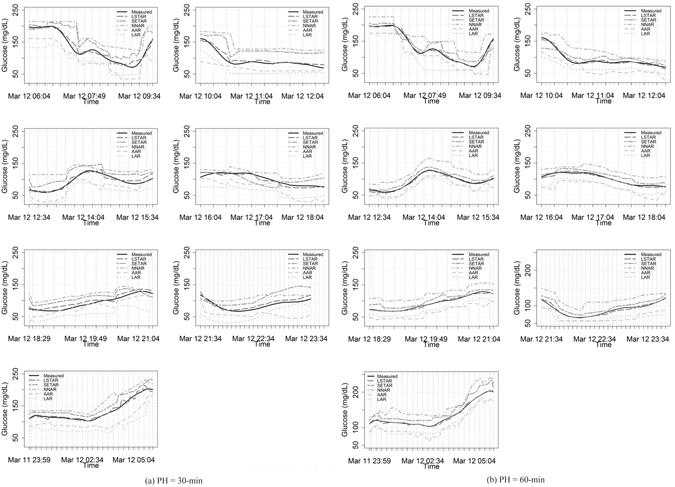
Profiles of measured and predicted glucose time series using AR models for a PH of 30 minutes ((**a**), from left to right: *morning*, *late-morning*, *noon*, *afternoon*, *early-evening*, *evening*, *night*) and a PH of 60 minutes ((**b**), from left to right: *morning*, *late-morning*, *noon*, *afternoon*, *early-evening*, *evening*, *night*).

We applied the models to test sets considering PH values of 30 minutes and 60 minutes. The results are presented in Tables 3 and 4 for a PH of 30 minutes and 60 minutes, respectively. For a typical patient with a high risk of both hyper- and hypo-glycemia, and landmark time intervals of high and low-moderate risk levels, the resulting average prediction errors for the LSTAR model were 6.02 (±0.38) mg/dL RMSE and 7.15 (±0.85) mg/dL RMSE for a PH of 30 minutes and 60 minutes, respectively. Plots of the measured and predicted glucose time series are presented in Fig. 5, corresponding to the glucose time series for the same patient with a high risk of both hyper- and hypo-glycemia, and alternate landmarks of high and low-moderate risk levels. Subsequently, CG-EGA is applied to evaluate the clinical accuracy of the glucose time series predictions and their utility for avoiding hypo- and hyper-glycemic events. According to the CG-EGA, each model presented the lowest performance for the landmarks with a high risk of hypoglycemia, while most zone A values were achieved by applying the LSTAR model (92.06% and 90.57% for a PH up to 30 minutes and 60 minutes, respectively). In order to more fully understand the results, we computed the RMSE and MAE values of the AR models for a PH of 30 minutes and 60 minutes when considering the seven input cases.

**Table 3:** Average prediction accuracy of AR models for PH of 30-minutes (RMSE – *Root Mean Squared Error*; MAE – *Mean Absolute Error*).

Cases	LAR		AAR		NNAR		SETAR		LSTAR	
	RMSE	MAE	RMSE	MAE	RMSE	MAE	RMSE	MAE	RMSE	MAE
*Low to moderate risk* ^(*1*)^										
	5.93 (1.93)	5.02 (1.65)	5.31 (1.96)	5.56 (1.54)	5.31 (2.47)	5.03 (2.21)	5.81 (1.44)	5.73 (1.52)	4.83 (2.28)	4.32 (2.51)
*High risk of hypoglycemia* ^(*1*)^										
	15.12 (1.41)	14.72 (2.02)	12.12 (1.43)	11.72 (2.12)	10.18 (1.46)	9.08 (1.58)	6.49 (0.77)	5.81 (1.31)	5.31 (1.96)	4.56 (1.54)
	16.65 (2.33)	14.24 (2.43)	13.65 (2.33)	13.24 (2.41)	10.06 (1.63)	8.25 (0.68)	7.04 (1.64)	6.93 (1.27)	5.63 (1.06)	4.98 (0.94)
	17.64 (1.27)	15.16 (0.82)	14.64 (1.27)	13.66 (1.62)	10.68 (5.60)	10.01 (4.84)	9.54 (3.19)	8.38 (2.33)	6.07 (2.61)	5.75 (1.97)
*High risk of hyperglycemia* ^(*1*)^										
	18.07 (3.43)	17.88 (3.21)	12.42 (4.18)	10.28 (3.81)	10.42 (3.18)	10.28 (2.81)	7.64 (0.93)	6.67 (0.93)	5.71 (0.74)	4.93 (0.93)
	18.44 (4.19)	18.27 (3.23)	13.62 (4.37)	11.746 (3.41)	11.62 (3.37)	11.46 (3.41)	8.83 (2.22)	7.67 (1.68)	5.74 (2.03)	5.05 (1.94)
	17.44 (4.13)	17.11 (1.76)	13.62 (4.37)	10.81 (3.41)	11.62 (4.31)	10.81 (3.28)	9.64 (3.22)	8.53 (2.15)	6.51 (3.28)	5.81 (2.51)

^(1)^Values are presented as mean (standard deviation).

**Table 4:** Average prediction accuracy of AR models for PH of 60-minutes: RMSE – *Root Mean Squared Error*; MAE – *Mean Absolute Error*.

Cases	LAR		AAR		NNAR		SETAR		LSTAR	
	RMSE	MAE	RMSE	MAE	RMSE	MAE	RMSE	MAE	RMSE	MAE
*Low to moderate risk* ^(*1*)^										
	7.43 (1.93)	6.04 (1.65)	7.01 (1.96)	6.56 (1.54)	6.11 (2.47)	5.03 (2.21)	7.15 (1.42)	6.83 (1.22)	6.01 (3.28)	5.81 (2.51)
*High risk of hypoglycemia* ^(*1*)^										
	18.54 (1.73)	17.92 (2.53)	13.14 (1.33)	12.72 (2.02)	10.88 (2.06)	9.38 (2.98)	9.25 (1.47)	8.84 (1.03)	7.31 (1.96)	6.56 (1.54)
	16.95 (2.93)	15.44 (2.75)	13.85 (2.83)	13.44 (2.71)	10.06 (2.61)	8.25 (1.98)	8.04 (1.62)	7.93 (1.75)	7.63 (1.06)	6.98 (0.94)
	17.41 (1.78)	15.86 (1.23)	14.84 (1.72)	13.96 (1.72)	11.81 (2.60)	10.51 (3.82)	8.31 (2.97)	7.83 (1.03)	7.68 (2.14)	6.92 (1.73)
*High risk of hyperglycemia* ^(*1*)^										
	17.07 (3.43)	17.88 (3.21)	13.62 (4.11)	11.38 (3.12)	10.42 (4.18)	10.28 (3.14)	8.64 (0.93)	7.67 (0.93)	6.71 (0.74)	5.93 (0.93)
	18.44 (3.19)	18.27 (3.03)	13.62 (4.37)	11.62 (3.19)	11.62 (4.37)	11.49 (3.81)	9.83 (2.12)	7.67 (1.83)	7.74 (2.03)	6.05 (1.94)
	17.84 (3.27)	16.21 (2.06)	13.82 (3.72)	11.95 (3.71)	11.42 (4.71)	10.15 (3.91)	9.04 (2.07)	8.03 (2.03)	7.51 (3.28)	6.81 (2.51)

^(1)^Values are presented as mean (standard deviation).

We observed that the LSTAR model achieved the lowest error values, 6.07 (±2.61) mg/dL RMSE and 5.75 (±1.97) mg/dL MAE for patients with glucose profiles at high risk of hypoglycemia, when compared to LAR, AAR, NNAR, and SETAR for a PH of 30 minutes (Table 3). Similarly, for a PH of 60 minutes, the LSTAR model still presented the lowest error values: 7.68 (±2.14) mg/dL RMSE and 6.92 (±1.73) mg/dL MAE (Table 4). In the case of time series with low and moderate risk, all models performed similarly well for a PH of both 30 minutes and 60 minutes.

According to the CG-EGA, each model presented the lowest performance in the hypoglycemia range, while most zone A values were achieved by applying the LSTAR model (90.15% and 89.72% for a PH up to 30 minutes and 60 minutes, respectively). In the cases of landmarks with low and moderate risk, all five models performed well even when the PH was increased to 60 minutes.

## Discussion

This study was conceived to investigate the potential use of nonlinear regime-switching AR models to predict the glycemic levels of T1DM patients in order to enable real-time decision support systems. The novelty lies in designing the predictive models based on the chaotic properties of the measured glucose time series, which were a posteriori related to the corresponding hyper- or hypo-glycemia risk levels for each patient. The main advantage of this approach is minimal patient intervention, avoiding the need to wear additional sensors or track daily events.

Low-dimensional input spaces have the advantage of requiring small time intervals for training the models, which is very important for real-time decision support systems. Although all models performed well in the euglycemic range, the most accurate predictions in the hypo- and hyper-glycemia ranges were achieved by the LSTAR models, demonstrating their superiority to the LAR, AAR, NNAR, and SETAR models for all input cases. For a PH of 30 minutes, the errors in the nonlinear models were higher for patients in the hypoglycemia range in the late-morning, afternoon, and evening landmark periods, while the lowest errors were observed for patients in the hypoglycemia range over the landmark time intervals corresponding to after-meal landmarks. Similarly, for a PH of 60 minutes, the lowest and highest errors were observed for patients in the hypo- and hyper-glycemia ranges in after-meal periods. This can be explained by a lower percentage of hypoglycemic events due to the diabetic patients undergoing insulin treatments during the monitoring period. However, the input cases included patients with a high-risk of hypoglycemia over all landmark time intervals. Without including exogenous information, the nonlinear AR models performed similarly to the support vector regression method^16^, which achieved errors of 6.03 mg/dL RMSE and 7.14 mg/dL RMSE for a PH of 30 minutes and 60 minutes, respectively, but required a longer time period for data processing and extensive effort from the patient. Prediction performance was not improved by adding insulin delivery and meal content information to the CGM data during sleeping periods^29^. Furthermore, the proposed AR models outperformed previous AR methods, which achieved errors of 18.78 mg/dL RMSE for a PH of 30 minutes^12^, 17.5 mg/dL RMSE for a PH of 30 minutes^14^, and 3.83 mg/dL MAPE for a PH of 30 minutes^30^. One exception is the approach presented in ref. 31, where the predictive performance was assessed on datasets with patients not included in the training set, resulting in errors of 43.9 mg/dL RMSE for a PH of 75 minutes.

In this study, the dimension of the training set was chosen based on physician expertise and remained constant for all experiments. We believe that an approach where the length of the time series used for training the models changes based on the GV and risk level of each time series would further improve prediction accuracy.

Simulation studies of glucose controllers for closed-loop systems have already been conducted by employing linear or linearized methods^32^ and nonlinear approaches^33^. Further work would include clinical data evaluation of nonlinear regime-switching AR models in conjunction with a controller designed to provide feedback on glucose regulation by delivering personalized levels of insulin.

## Methods

### Dataset

The data was collected by monitoring 17 patients with T1DM for between four and seven days (average, 5.73 ± 1.03) under free-living conditions. Patients wore a real-time CGM system developed by Medtronic and were enrolled in the Clinic of Diabetes, Nutrition and Metabolic Diseases at the “Pius Brinzeu” Emergency Hospital of Timisoara, Romania. All medical procedures were performed in accordance with relevant guidelines and regulations. The study protocol and informed consent forms were reviewed and approved by the Ethics Committee of the Emergency Hospital Timisoara. All patients signed an informed consent form.

The CGM system reported an average s.c. glucose value every five minutes. The observation period was divided into seven landmark time intervals: *morning* (*M*) from 6 am to 10 am, *late-morning* (*LM*) from 10 am to 12:30 pm, *noon* (*N*) from 12:30 pm to 4 pm, *afternoon* (*AN*) from 4 pm to 6:30 pm, *early-evening* (*EE*) from 6:30 pm to 9:30 pm, *evening* (*E*) from 9:30 pm to 12:00 am, and *night* (*N*) from 12:00 am to 6 am. The time intervals respected daily schedules and were valid for all datasets. Hypoglycemia was defined as an event in which at least two consecutive s.c. glucose values were below 70 mg/dL, while hyperglycemia was defined as an event in which at least two consecutive s.c. glucose values were above 180 mg/dL.

### Input cases

We considered generic input cases to differentiate patients based on their risk level and GV in the landmark glucose time series. The first case included patients who were at low and moderate risk of both hypo- and hyper-glycemia over the entire monitoring period. Case 1 contained two patients. The second, third, and fourth generic cases included patients at high-risk of hypoglycemia in at least one of the *morning*, *early-noon*, or *evening* landmark time intervals; in at least one of the *late-morning*, *afternoon*, or *late-evening* landmark time intervals; and in the *night* landmark time interval. The second case included two patients, while the third and fourth cases included three patients each. The next three cases were similar to the previous ones, but considered patients with a high-risk of hyperglycemia in the respective landmark time intervals, including two patients in the fifth and sixth cases, and three patients in the eighth case.

### Chaotic properties

We analysed the chaotic properties of the glucose time series through various computations, including embedding space, correlation dimension, and Lyapunov exponents. The Lyapunov exponent, denoted λ, is an averaged exponent that determines a divergence rate. It is estimated using the slope obtained by performing the linear regression $S(t)=\lambda \cdot t \sim \mathrm{log}(\delta (t))/\delta (0)$ on *t*, where *δ*(0) is the distance between two Takens’ vectors in the embedding space, $\delta (t) \sim \delta (0)\cdot \exp (\lambda ,t)$ is the distance after a time *t* between the two vectors, and *S*(*t*) is estimated by averaging the divergences of several reference points.

### Glucose predictive models

The CGM measurements represent a series of chronological observations $\{{x}_{1},{x}_{2},\cdots ,{x}_{t},\cdots \}$ recorded at equidistant time points, where *x*_*t*_ represents the measurement at *t*-th observation. Time series observations are transformed into phase-space vectors $\{{z}_{1},{z}_{2},\cdots ,{z}_{t},\cdots \}$ via time delay embedding. When considering chaotic systems, modelling the dynamics of the system can be accomplished by modelling the dynamics of corresponding points in the phase-space^34^.

### Phase-space reconstruction

Considering the theorem presented by Takens^35^, we postulate that it is possible to reconstruct the original time series by using time delay embedding vectors, where the delay vectors consist of scalar measurements of the system’s state. The delay vectors ${z}_{t}=[{x}_{t},{x}_{t+\tau },\cdots ,{x}_{t+(m-1)\tau }]$ are used to generate the phase-space, where *m* is the embedding dimension and *τ* is the time delay. Therefore, it follows that the dynamic properties of the state-space system are preserved through the embedding transformation. Consequently, time series are described by their specific embedding dimensions and time delays. The time delay *τ* is defined as the average mutual information of the corresponding glucose time series between *x*_*i*_ and *x*_*i*+*τ*_ as follows:

1 $$\tau =\sum _{ij}{p}_{ij}(\tau )log({p}_{ij}(\tau )/({p}_{i}(\tau ){p}_{j}(\tau )))$$

where *p*_*i*_(*τ*) is the probability that the time series *x*_*i*_ has a value inside bin *i* of the data histogram and *p*_*ij*_(*τ*) is the probability that *x*_*i*_ belongs to bin *i* and *x*_*j*−*τ*_ belongs to bin *j*. The embedding dimension *m* is defined as the fraction of false neighbours over the total number of neighbours of the corresponding time series. The values of embedding dimension and time delay are used to transform the time series into the phase-space vectors *z*_*t*_, expressed as:

2 $${z}_{u}=(\begin{array}{c}{z}_{0}\\ {z}_{1}\\ \begin{array}{c}{z}_{2}\\ \vdots \end{array}\end{array})=(\begin{array}{ccc}\begin{array}{c}{x}_{0}\\ {x}_{1}\end{array} & \begin{array}{c}{x}_{\tau }\\ {x}_{1+\tau }\end{array} & \begin{array}{cc}\cdots  & {x}_{(m-1)\tau }\\ \cdots  & {x}_{1+(m-1)\tau }\end{array}\\ \begin{array}{c}{x}_{2}\end{array} & \begin{array}{c}{x}_{2+\tau }\end{array} & \begin{array}{cc}\cdots  & {x}_{2+(m-1)\tau }\\ \vdots  & \,\end{array}\end{array})$$

where *z*_*u*_ is the matrix form of the phase-space vectors.

#### Prediction in phase-space

We consider the discrete-time dynamic system generated by the mapping function:

3 $${z}_{t+1}=F({z}_{t})=\sum _{d=1}^{D}c(d,t){\phi }_{d}({z}_{t})+{\varepsilon }_{t}$$

which is a linear combination of *D* possible nonlinear functions *φ*_*d*_, with *c*(*d*, *t*) representing their coefficients. Typically, the coefficients are computed via functional approximation, such as the least squares method. The AAR model is defined by the following expression^36^:

4 $${z}_{t+1}=\mu +\sum _{i=1}^{m}{s}_{i}({z}_{t-(i-1)\tau })$$

where *s*_*i*_ are smoothing functions represented by penalized cubic regression splines. The NNAR model with linear input and *g* as the activation function follows the expression:

5 $${z}_{t+1}={\beta }_{0}+\sum _{j=1}^{q}{\beta }_{j}g({\theta }_{0j}+\sum _{i=1}^{m}{\theta }_{ij}{z}_{t-(i-1)\tau })+{\varepsilon }_{t}$$

where *q* is the number of hidden units. The SETAR model is defined in delay embedding space as follows:

6 $${z}_{t+1}=\{\begin{array}{c}{\theta }_{1}+{\theta }_{10}{z}_{t}+{\theta }_{11}{z}_{t-\tau }+\cdots +{\theta }_{1L}{\theta }_{t-(L-1)\tau }+{\varepsilon }_{t+1}{Y}_{t}\le th\\ {\theta }_{2}+{\theta }_{20}{z}_{t}+{\theta }_{21}{z}_{t-\tau }+\cdots +{\theta }_{2H}{\theta }_{t-(H-1)\tau }+{\varepsilon }_{t+1}{Y}_{t} > th\end{array}$$

where *T*_*t*_ is a threshold variable in $\{{z}_{t},{z}_{t-\tau },\cdots ,{z}_{t-(m-1)\tau }\}$, which can be defined by the threshold delay $\delta \in \{0,\cdots ,m-1\}$, because ${Y}_{t}={z}_{t-\delta \tau }$, or as a linear combination of lagged time series values ${Y}_{t}={\beta }_{1}{z}_{t}+{\beta }_{2}{z}_{t-1}+\cdots +{\beta }_{m}{z}_{t-(m-1)\tau }$. The regime at a particular *t*ime *t* can be determined from the observed data in the vicinity of the threshold value. The model is linear within a regime; however, it is capable of moving between regimes as the threshold changes. If the threshold is replaced by a logistic function: 0 < *G*(*T*_*t*_) < 1, then depending on the transition variable *Y*_*t*_, the LSTAR model in delay embedding space follows the expression:

7 $${z}_{t+1}=({\theta }_{1}+\sum _{i=0}^{L}{\theta }_{1i}{X}_{t-i\tau })(1-G({Y}_{t}))+({\theta }_{2}+\sum _{i=0}^{H}{\theta }_{2i}{X}_{t-i\tau })(1-G({Y}_{t}))$$

The first order logistic function depends on a location *c* and scale *γ*, where $G({z}_{t};\gamma ,c)={(1+{e}^{-\gamma ({Y}_{t}-c)})}^{-1}.$ This model achieves a more gradual transition between regimes, with *c* influencing the threshold between regimes and *γ* determining the smoothness of the change.

### Time series reconstruction

We extracted the time series from the univariate *z*_*u*_ matrix of predictions in the phase-space by retaining the first column and the last *τ* rows of the matrix. A matrix *z*_*u*_ of dimension *T* × *m* generates *T* + (*m* − 1)*τ* time series observations: ${x}_{i}\in \{{z}_{u}(1,i),{z}_{u}(k,T-j)\}$, where $0\le i < T,0 < j\le \tau ,1\le k < m.$

## Conclusions

In the context of diabetes-related risk management, nonlinear regime-switching autoregressive models requiring minimal patient intervention were proposed for predictive modelling of glucose dynamics. Estimation of model parameters was based on the chaotic properties of the glucose time series for each patient. The models provided faster prediction with higher accuracy when compared to previous time series predictive models. Fast prediction can enable real-time decision support systems, allowing the patient to make therapeutic decisions based on future glucose levels, and decreasing the risk of hypo- and hyper-glycemic events.
